# Modified Mycotoxins and Multitoxin Contamination of Food and Feed as Major Analytical Challenges

**DOI:** 10.3390/toxins15080511

**Published:** 2023-08-19

**Authors:** Ksenija Nešić, Kristina Habschied, Krešimir Mastanjević

**Affiliations:** 1 Institute of Veterinary Medicine of Serbia, Food and Feed Department, Smolućska 11, 11070 Beograd, Serbia; 2 Faculty of Food Technology Osijek, Josip Juraj Strossmayer University of Osijek, F. Kuhača 20, 31000 Osijek, Croatia; kmastanj@gmail.com

**Keywords:** masked mycotoxins, multiple mycotoxins, mycotoxicological analysis

## Abstract

Mycotoxins, as natural products of molds, are often unavoidable contaminants of food and feed, to which the increasingly evident climate changes contribute a large part. The consequences are more or less severe and range from economic losses to worrying health problems to a fatal outcome. One of the best preventive approaches is regular monitoring of food and feed for the presence of mycotoxins. However, even under conditions of frequent, comprehensive, and conscientious controls, the desired protection goal may not be achieved. In fact, it often happens that, despite favorable analytical results that do not indicate high mycotoxin contamination, symptoms of their presence occur in practice. The most common reasons for this are the simultaneous presence of several different mycotoxins whose individual content does not exceed the detectable or prescribed values and/or the alteration of the form of the mycotoxin, which renders it impossible to be analytically determined using routine methods. When such contaminated foods enter a living organism, toxic effects occur. This article aims to shed light on the above problems in order to pay more attention to them, work to reduce their impact, and, eventually, overcome them.

## 1. Introduction

Mycotoxins are already-known natural products of low molecular weight that are synthesized as secondary metabolites of filamentous fungi, usually *Fusarium*, *Penicillium*, and *Aspergillus*, and are often found as contaminants in food and feed [1]. They represent a toxic and chemically heterogeneous group, classified together for their ability to damage human and animal health (mycotoxicosis) and even cause fatal outcomes. They were named in 1962 after an unexpected animal health crisis near London, England, in which about 100,000 turkeys died from the mysterious X disease, which was linked to peanut meal contaminated with aflatoxins, secondary metabolites of *Aspergillus flavus*. This motivated numerous studies and expanded the number of mycotoxins discovered. In the period from 1960 to 1975, a large number of scientists engaged in well-funded research into new mycotoxins, which is why these years are also known as the mycotoxin rush. Today, there are about 400 different compounds bearing this name, and among them, there are a dozen that attract special attention because they pose a threat to human and animal health [2].

Mycotoxins can be synthesized by fungi directly in the field, already on the plants during the growing season (if they remain in the agricultural products at the time of their collection), or produced during storage or technological processes. Their presence is related to the fungal genus or species, the agronomic practices, the type of crop, and the harvesting, handling, and storage conditions. However, they are considered one of the foodborne risks that correlate with climatic changes. In this context, it is important to keep in mind that the two most important factors affecting the life cycle of all microorganisms, including mycotoxic molds, are water availability and temperature. In general, the amount of toxins synthesized depends on several factors: physical factors such as the temperature, the relative humidity, the moisture of the matrix, the water activity (aw), and the degree of mechanical damage to the grains; chemical factors such as the oxygen and carbon dioxide content, the composition of the substrate, and the presence of pesticides and fungicides; and biological factors such as the plant variety affected, the presence of stressors, the influence of insects, and the spore load [3]. Anything that can influence these factors can directly or indirectly change the mycotoxicological situation.

Based on these facts, weather extremes have been found to influence the occurrence of mycotoxins in food and feed. For example, years with regional weather conditions described as extreme (high temperatures, lack of precipitation, and pronounced drought) are prone to the occurrence of aflatoxins (AFs) in crops. This was reported in 2021, when the highest recorded prevalence of AFs in corn samples was 84% in Serbia and 40% in Croatia. Earlier reports for corn originating from the 2018–2020 cropping period are much more favorable, showing contamination in 10% of Serbian and 20% of Croatian samples. [4]. The problem of the presence of AFs in cereals from Serbia was the cause of the elevated levels of aflatoxin M1 in milk and dairy products a few years earlier [5], while the risk assessment in Croatia for the period 2016–2022 indicated that there is cause for concern in autumn and winter, especially for consumers of large quantities of milk [6]. The occurrence and contamination level of Fusarium mycotoxins in the maize samples analyzed in the study from the same region for the period 2018–2022 varied from year to year and might be related to climatic conditions. In this study, FUMs were identified as the most prevalent contaminants in maize in Serbia and Croatia. When analyzing the ten-year results from 2012 to 2022, the highest levels of DON and ZEN were found in maize samples from 2014. This could be due to the extreme rainfall observed in both countries in that year [7].

Such findings are not surprising, given the report of the Intergovernmental Panel on Climate Change (IPCC) [8], which states that the effects of global warming have already been observed and that this has already affected ecosystems and some of the services they provide. Climate-related risks to health, livelihoods, food security, water supply, human security, and economic growth are projected to increase with 1.5 °C of global warming and to be worse with a 2 °C increase. The IPCC further summarizes that there is a high probability of extreme heat in most populated regions and an increase in average temperatures in most terrestrial and marine regions, while it predicts a medium probability of heavy precipitation in several regions and the likelihood of droughts and precipitation deficits in others. In the face of these predictions, the safety of food (and feed) will be compromised in a number of ways [9], and only one of these is infestation with toxic molds and contamination with their secondary metabolites—mycotoxins.

Various preventive measures and control strategies, as well as treatments, can be tried [10,11,12], although the absolute elimination of these natural substances is not possible. In this struggle, the monitoring of food and feed and the timely determination of the nature and quantity of these contaminants [13] have a very important place in controlling the harmful effects on living organisms caused by their consumption. However, even on this path, despite modern techniques and growing knowledge on the subject, there are some obstacles. The aim of this paper is to contribute to the elucidation of the two most common stumbling blocks and reasons for the inconsistency between analytical data and practical events: multitoxin contamination and the presence of modified (so-called masked) mycotoxins.

## 2. Multitoxin Contamination

Mycotoxins are a huge global food safety concern, especially in light of recent estimates that they contaminate 60–80% of the food produced worldwide [14] and that this group consists of hundreds of identified species. Among them, aflatoxins (AFs), ochratoxin A (OTA), fumonisins (FUMs), deoxynivalenol (DON), patulin (PAT), nivalenol (NIV), the T-2 toxin (T-2), the HT-2 toxin (HT-2), and zearalenone (ZEA) and its derivatives are of greatest concern as they are common and frequently detected in nature [15]. Therefore, many countries have set individual limits for various foods intended for human and animal consumption [16]. Current legislation refers to the assessment of risks posed by individual mycotoxins and, initially, by the metabolites of mycotoxins together with the parent compound. However, they do not take into account the multiple dynamics and potential interactions between co-occurring groups of mycotoxins [17].

Adding to all of the above the increasingly frequent extreme climatic events, to which fungi and their secondary metabolism are highly susceptible, the real mycotoxicological picture is often far more complex than typical patterns and goes far beyond one-sided viewpoints. Since a single fungal species can produce various mycotoxins and fungi of different genera may infect the same crops, the co-occurrence of several mycotoxins and simultaneous exposure in the total diet are even more likely. Such exposure to a combination of mycotoxins can cause the same effects or lead to a combination of different side effects. As stated by van den Brand et al. [18], when assessing the combined risk of co-occurring mycotoxins, this should be taken into account.

A significant analysis of the literature data on the presence of mycotoxins in foods derived from cereals in Europe and their natural co-occurrence was provided by Palumbo et al. [19]. The database for the presence of mycotoxins in cereals included twelve crop groups: corn, wheat, barley, oat, rye, sorghum, triticale, buckwheat, spelt, rye, millet, and soy. Mycotoxins were commonly noted in wheat and maize, with the highest occurrence of FUMs, DON, AFs, and ZEA. The highest content of FUMs (B1 + B2) was reported in corn (both for feed and food) and was above legal maximum levels (MLs). Similar results were found for DON in food, whose maximum concentrations in corn, wheat, oats, and barley exceeded the MLs. The co-occurrence of two or more mycotoxins was reported in 54.9% of the total records. Co-contamination of DON is usually observed with FUMs in corn and ZEA in wheat, while the combination of DON + NIV and DON + T2/HT2 is often reported in barley and oats. Some publications, analyzed in a Portuguese study [20], reported the co-occurrence of mycotoxins in 75% of foods derived from cereals. A similar percentage for the three mycotoxins ZEA, DON, and NIV was noted in 74% of wheat samples from Brazil sampled in 2009 and 12% originating in 2010, with precipitation during flowering or harvest periods explaining the difference between these results [21]. In Korea, co-occurrence of mycotoxins with glucoside conjugates has been reported with a maximum of 49% [22]. Another Korean study [23] investigated the levels of 13 mycotoxins in five types of commercial cereals (brown rice, maize, millet, sorghum, and mixed grains) and showed that *Fusarium* mycotoxins (FUMs, DON, NIV, and ZEA) were more frequently and simultaneously detected in all cereals and with higher mean concentrations than other toxins, like ochratoxin A and aflatoxins. Joshi et al. [24] examined multi-mycotoxin contamination in Nepalese maize, and they found out that all the samples contained at least a couple of mycotoxins, while more of them were found in 87% of the examined samples. The most frequently encountered binary, ternary, and quaternary contaminants were DON + AFs, AFs + FUMs + DON, and AFs + FUMs + ZEA + DON, respectively. An Algerian survey on 120 grain samples from markets (corn, wheat, barley, and rice) was performed to evaluate the presence of 15 mycotoxins. The test results showed that 65% of the samples (78 of them) were contaminated with some of the mycotoxins, while 50% contained between three and nine different types, with deoxynivalenol, the T-2 toxin, beauvericin, and citrinin being the most commonly found [25].

As reviewed by Tolosa et al. [26], animal feed is particularly vulnerable to contamination by multiple mycotoxins as it is a mixture of several raw ingredients, so it already becomes the rule, not the exception, while the transfer of mycotoxins from animal feed to food of animal origin is often demonstrated. According to Gruber-Dorninger et al. [27], out of 74,821 samples of feed and feed raw materials collected from 100 countries from 2008 to 2017, 64% proved positive for at least two mycotoxins. The most frequently observed mycotoxin mixtures were combinations of DON, ZEA, and FUMs, as well as FUMs and AF B_1_. According to their extensive global survey, they concluded that (co-)contamination of animal feed with mycotoxins is common, regionally defined, and partially driven by climate and weather.

Considering the results of multi-mycotoxin monitoring studies in Europe, a large percentage of feed has been found to contain more than one mycotoxin. Belgium’s Monbaliu et al. [28] reported that 75% of samples (sow feed, wheat, and maize) were contaminated with more than one type of mycotoxin, while type B trichothecenes and FUMs occurred most often. Up to 90% of the 1384 samples of raw materials and products for animal feed (complete feed and components like small grain, corn, and corn silage) from Poland, analyzed by Kosicki et al. [29], showed to contain DON and ZEA. They commonly found the combination of DON, T-2, and HT-2; ZEA, T-2, and HT-2; DON, T-2, HT-2, and ZEA; and DON and FUMs in maize. According to Romanian authors Stanciu et al. [30], 42% of the 116 cereal samples tested contained between two and five mycotoxins, with enniatin B being the most prevalent, followed by DON and ZEA. Castaldo et al. [31] dealt with the presence of 28 mycotoxins in 89 pet food samples from Italy. They reported mycotoxin contamination in 99.9% of the samples, with all the positive samples showing co-occurrence of mycotoxins and the simultaneous presence of up to 16 mycotoxins per sample.

Finally, an increasingly topical issue is the occurrence of so-called “emerging mycotoxins”. This group consists of currently non-regulated mycotoxins produced by *Fusarium* spp., which include beauvericin (BEA), enniatins (ENNs), and fusaproliferin (FUS). Permitted levels have not been set for these mycotoxins as there are not enough data regarding their toxicity, occurrence, and contamination levels [32]. However, beauvericin (BEA) and enniatins (ENNs) are found in cereals to a large extent, although the pattern of contamination varies between crops and the contents strive to be higher in cold climates [26]. Their co-occurrence was observed in 47% of the samples, as stated by Tolosa et al. [33]. Also, the co-contamination of 87% of maize silage samples with ENNs, BEA, and other *Fusarium* mycotoxins, such as DON and NIV, was reported by Reisinger et al. [34], which Krizova et al. reviewed later [35].

Regular monitoring of different mycotoxins and the collection of data on their presence and co-occurrence, with details of the location of the crops, as well as the availability of this information, should be a priority. By adding knowledge about weather forecasting and plant phenology, it is possible to build predictive models to estimate mycotoxin risk levels with great confidence [36]. This would be highly beneficial for different stakeholders: farmers; the feed and food industry, including collectors, millers, and processors; food safety authorities; and above all, final consumers [3]. Table 1 summarizes information on the presence of multiple mycotoxins in different feed and food samples, their incidence, and data sources. 

## 3. Modified Mycotoxins

The label for modified mycotoxins refers to masked and bound mycotoxins and mycotoxin metabolites. The name “masked” comes from the fact that, until recently, they were not detected during routine feed and food tests, and there were no maximum limits in force, so they were only occasionally discovered during the analysis of original forms of mycotoxins. These native, unchanged forms are called “parent compounds”. Modified mycotoxins can be described as metabolites of the parent mycotoxins made in the plant by plant enzymes as a result of their defensive reaction to mold infestation, or they can be synthesized by the molds themselves. They can also have an abiotic origin due to a chemical reaction between the parent toxin and the matrix that occurs during food processing. Some research has also indicated the possibility that modified mycotoxins are produced during metabolic processes in animals and humans, but these conjugates are extremely unlikely to play any role in feed or food and are therefore not regarded as masked mycotoxins [36].

The first modified mycotoxin discovered before they were named “masked mycotoxins” was aflatoxin M1 (excreted in milk), which is a metabolite of aflatoxin B1 formed by hydroxylation in the organisms of animals that consume feed contaminated with this mycotoxin. Following this discovery, another compound that is ZEA-derived was discovered in the 1980s. However, the revelation of different forms of mycotoxins has only intensified in recent years [37]. As *Fusarium* infestations usually happen in the field, their mycotoxins are the most dominant targets for conjugation with sugars, amino acids, or sulfate groups in the conversion process carried out by host plants. Therefore, the most frequently modified mycotoxins belong to the family of fusariotoxins and are -linked glucose-conjugates of deoxynivalenol (DON3G), zearalenone (ZEA14G, α-ZEL14G, β-ZEL14G), zearalenone-14-sulfate (ZEA14S), nivalenol (NIV3G), fumonisin esters, T-2/HT-2 (T-2 and HT2G), and a few others (Table 2). The transformation of other toxins, such as ochratoxin A, patulin, and destruxins, by plants has also been described [38].

As summarized by Freire and Sant’Ana [37], the predominant mycotoxins and their modified forms in feed and food samples are DON with DON-3-glucoside; DON-Hexitol; DON-S-cysteine; DON-S-cysteinyl-glycine; DON-glutathione; DON-di-hexoside; DON-2H-glutathione; DON-malonylglucoside; 15-acetyl-DON-3-glucoside; 3-Acetyl-DON; DON-3-sulfate; DON-15-sulfate; 3-epimer-DON; norDON A, B, and C; norDON-3-glucoside A, B, C, and D; DON-3-glucoside-lactone; de-epoxy DON; DON-glucuronide; de-epoxy DON-3-sulfate; de-epoxy DON-15-sulfate; ZEN with ZEN-16-O-β-glucoside; ZEN-14-O-β-glucoside; α-zearalenol; β-zearalenol; α-zearalenol-glucoside; β-zearalenol-glucoside; ZEN-4-glucoside; ZEN-4-sulfate; malonyl-glucosides (ZEN-MalGlc, α-ZEN-MalGlc, β-zearalenol-MalGlc); di-hexose-(ZEN-DiHex, α-zearalenol-DiHex, β-zearalenol-DiHex); hexose–pentose disaccharides (ZENHexPent, α-zearalenol-HexPent, β-zearalenol-HexPent); tri-hexose conjugate (β-zearalenol-TriHex); α-zearalenol-Sulfate; α-zearalenol; β- zearalenol; OTA with ochratoxin α; 4S-hydroxyochratoxin A; 4R-hydroxyochratoxin A; hydroxyochratoxin A-β-glucoside; ochratoxin A methyl ester; ochratoxin α amide; 14-decarboxy-ochratoxin A; ochratoxin A mono- and disaccharide esters; T-2/HT-2 with HT2 toxin-3-glucoside; T-2 toxin-α-glucoside; T-2 toxin-β-glucoside; 15-acetyl-T2-tetraol-glucoside; hydroxy-HT2-glucoside; hydroxy-HT2-malonyl-glucoside; T2-triol-glucoside; dehydro-HT2-glucoside; HT2-diglucoside; HT2-malonyl-glucoside; 3-acetyl-HT2; 3-acetyl-T2; feruloyl-T2; HT2-sulfate and FUM with hidden fumonisins; N-(carboxymethyl) fumonisin B1; N-Acyl hydrolyzed fumonisin B1; and bound hydrolyzed fumonisins.

Although most mycotoxin modifications arise from the defense system of plants, the metabolism of microorganisms, and even human and animal organisms as an attempt to detoxify the parent mycotoxin, some of them still exhibit toxicity, even if it is decreased. However, a major concern is the rehabilitation of the parent mycotoxin in the human or animal gastrointestinal tract. In addition, some of these formed compounds can exhibit higher toxicity if they have a higher bioavailability and bioaccessibility than the parent mycotoxin [37]. For example, studies on fumonisins show adverse effects after food intake, even at low levels. This indicates the contribution of the present derivatives. It is assumed that FUMs bind to carbohydrates or proteins in food during thermal processes and convert back to the parent mycotoxin after intake. In order to examine the toxic effects of primary mycotoxins and their modifications, in vitro and in vivo studies have been conducted [37,38].

Nevertheless, toxicological data on modified mycotoxins are still limited [40]. Glucoside conjugates of trichothecenes can be hydrolyzed to toxic compounds during digestion and may pose a safety risk. On the other hand, some studies on the occurrence and toxicity of modified mycotoxins, such as DON-3G, have shown that mycotoxin conjugates have a lower toxicity potential due to lower absorption in the gastrointestinal tract [41,42,43]. There are two types of reactions responsible for the chemical changes in toxins. Phase I reactions consist mainly of hydrolysis, reduction, and oxidation, while phase II reactions are characterized by conjugation. Formation occurs by conjugation with polar compounds, most commonly sugars such as glucose and modified glucose, but also other groups such as sulfate and glutathione. Fumonisins appear to be covalently and non-covalently bound to the matrix, so that some forms are physically trapped. The metabolites formed, often not as toxic, are bound to macromolecules or stored in the vacuole in soluble form. Although modified mycotoxins often have lower intrinsic toxicity, they can be reactivated during metabolism in mammals. In particular, the polar group can be cleaved (e.g., by intestinal bacteria), releasing the primary mycotoxin [39,44].

DON-3G is the most reported and described modified mycotoxin, although there are other modifications of DON (Table 2). DON-to-DON-3-G bio-conversion presumably takes place during the germination stage of the plant due to higher and more easily available glucose levels [45]. Formation was shown to be influenced by the parent toxin concentration, as higher DON levels were reported to affect the efficiency of DON-to-D3G conversion. This limits plants’ glycosylation capacity [46]. Compared to modified DON forms, research regarding the biotransformation of other type B trichothecenes is scarce. Nevertheless, a few studies described the synthesis of FUS-X and NIV glucosyl-conjugates in wheat [47,48,49]. The biotransformation of type A trichothecenes has been studied in barley and oats for T-2 and HT-2 toxins [50], describing glycosyl- and/or malonyl-conjugates and the quick phase I conversion in plants of T2 into HT2 through deacetylation. At the same time, the HT2 formed was further metabolized to HT2-3G by glycosylation. Zearalenone undergoes both phase I and phase II in plants with the formation of glucoside and sulfate conjugates of ZEA and its phase I metabolites-and-ZEL [46]. Several modified forms of fumonisins have been reported by now [51,52]. This is probably due to the impact of food and feed processing throughout the production chain. Unlike the modified trichothecenes and zearalenone, these conjugates are synthesized by the fungus during propagation on a plant substrate rather than by the plant itself [53]. Not many papers have been published on the possible biotransformation of OTA in plants, but several forms have been described (Table 2).

Regarding the presence of modified mycotoxins, as shown in Table 2, they are usually found in cereal-based food and feed [39]. Among the group of masked mycotoxins occurring in feed, ZEA-14-sulfate and DON-3-glucoside are the most common. ZEA-14-sulfate is a natural *Fusarium* metabolite, and after ingestion by animals, as it is easily hydrolyzed, ZEN is produced, causing an estrogenic response typical of the parent mycotoxin, especially in pigs, the most sensitive species. In contrast, the glucosides resist hydrolysis and are, therefore, not active [46]. Based on Broekaert et al.’s [54] literature summary of 13 research articles with a random sampling strategy, the incidence of trichothecenes and zearalenone modified forms in unprocessed cereals was up to 80% in the tested samples. Brya et al. [55] analyzed 88 maize products from the Polish retail market and concluded that more than 77% of the samples contained free and hidden fumonisins at concentrations above the LOQ. Furthermore, 51 coffee samples from France, Germany, and Guatemala were analyzed for the presence of OTA and its thermal isomer 2′R-ochratoxin A (2′R-OTA), and it turned out that 35% of the samples showed contamination with 2′R-OTA in a quantifiable amount [56].

Consuming contaminated feed and food results in exposure to both the parental compound and the modified form. This is why a combined exposure assessment needs to be conducted. Thus, referring to the limited data available, EFSA considers an additive effect of the metabolite, assigning the same toxicity as the respective parental compounds [44]. Compared to the presence of parent compounds only, the additional contribution of modified mycotoxins estimated by EFSA represents 100% for zearalenone, 60% for fumonisins, 30% for DON and nivalenol, and 10% for trichothecenes. Thus, the inclusion of ZEA modified forms represents a doubling of exposure, and a significant rise is also estimated for fumonisins. Generally, combined exposure to variable modified mycotoxins with possible synergistic or additive effects depicts a further aspect to be considered in risk assessment and requires an appropriate evaluation methodology such as that used in pesticide mixtures [39].

## 4. Laboratory Detection

Since the discovery of aflatoxins, various mycotoxins have been determined over time, belonging to several groups of toxins, and in parallel, analytical methods for their detection and quantification have been developed. Initially, mycotoxicological tests were performed for individual toxins or their families (aflatoxins, trichothecenes, fumonisins, ochratoxins, zearalenones). The analytical pathway was provided by molds that were present or had the highest probability of contaminating a given matrix and producing mycotoxins. In the course of time, various analytical techniques have been used, especially chromatographic ones: thin-layer chromatography (TLC), high-performance liquid chromatography (HPLC) combined with different detectors (ultraviolet—UV, fluorescence—FLD, diode array—DAD), liquid chromatography coupled with mass spectrometry (LC–MS), liquid chromatography-tandem mass spectrometry (LC–MS/MS), and gas chromatography-tandem mass spectrometry (GC–MS/MS) [57]. However, enzyme-linked immunosorbent assay (ELISA) [58] and lateral flow (LFD) [59] were used for rapid analysis.

Later, high-resolution mass spectrometry and biosensors were developed that allowed the relatively simultaneous detection of multiple toxins. This ensured the control of a larger number of possible contaminants and allowed a wider approach, as well as the detection of previously unknown potential hazards [60]. While immunochemical procedures are quite specific for a particular mycotoxin or group of close mycotoxins, up-to-date mass spectrometers are aimed at detecting structurally diverse mycotoxins in a single method. Antibody-based techniques and mass spectrometry are often combined (e.g., immunoaffinity column cleanup followed by LC-MS/MS), which provides very high selectivity. However, there are still some obstacles and shortcomings in analytical techniques: a wide range of chemical structures in which mycotoxins can be named, co-occurrence of mycotoxins, problems in detecting low mycotoxin contamination, multiplex food matrices in which mycotoxin contamination occurs, and complicated extraction methods [61].

From the above points of view, the most favorable and most widely used technique today is LC or HPLC coupled with tandem mass spectrometry detectors (LC-MS/MS), as it allows accurate, precise, and reliable confirmation of certain mycotoxins at low concentrations, even in complex matrices. It provides a simultaneous determination of more mycotoxins with various chemical structures [62]. A commonly used tool for the determination of mycotoxins is therefore the direct, indirect, competitive, or sandwich ELISA [63,64], which allows rapid and efficient screening with a simple sample pretreatment. To support field analysis, a transduction system with appropriate molecular recognition elements was introduced. In this sense, distinct analytical procedures have been developed for the detection of different mycotoxins: AFs, OTA, FUMB1, and trichotecenes [65]. Since the number of matrices tested is limited, the precision of the method can be minimized at low levels. In addition, the presence of matrix interferences and structurally similar mycotoxins may alter antibody binding and, consequently, mycotoxin quantification [61,66].

As emphasized by Agriopoulou et al. [67], crucial steps are the extraction procedure, the purification process, and the chromatographic separation, which should be regularly determined in order to significantly reduce matrix effects [68]. Inadequately pretreated samples can affect the sensitivity of mycotoxin detection and contribute to an overestimation of the results, as matrix effects can interfere with the interpretation of the results, especially in immunoassay-based methods with color compounds [69]. Both the matrix and the analyte determine the outcome, so the use of HPLC after immunoaffinity purification should be validated for each mycotoxin/matrix conjunction [70]. Furthermore, the co-elution of matrix components in LC-MS procedures suppresses or enhances chromatographic signals [68]. In the extraction step, solvents are used to remove mycotoxins from the contaminated food and feed samples. The choice of solvents as well as the extraction protocol contribute significantly to the output of the extraction. A suitable extraction solvent is one that removes only the mycotoxins from the sample with the highest efficiency. Prior to analysis, a mandatory cleaning step and the concentration of the sample are performed. Purification prior to the extraction procedure helps to increase selectivity by contributing to the further elimination of the interfering source from the matrix [71]. Table 3, following Agriopoulou et al. [67], summarizes the extraction protocols and solvents with their advantages and limitations.

### 4.1. Detection of Modified Mycotoxins

Modified mycotoxins are particularly challenging since their definition implies that the analysis of the mycotoxin content in the samples containing these compounds leads to their underestimation. Masked mycotoxins may escape analysis due to altered physicochemical attributes of their molecules, resulting in modified chromatographic behavior, modification of the epitope recognized by the antibodies used for detection, or reduced extraction efficiency caused by increased polarity when using a less polar solvent to extract non-modified mycotoxins. The bound mycotoxins completely escape conventional analysis. All these effects result in an underestimation of the total mycotoxin content in the sample. Modifications of mycotoxin molecules, which can act reductively or even abolish toxicity, could result in an obvious overestimation of mycotoxin levels. This is often the case when the analytical method detects the mycotoxin derivative together with the parent molecule but does not provide information regarding the analytical signal originating from a less toxic or non-toxic form. This is especially relevant for methods based on an antigen–antibody linkage reaction, as the epitopes recognized by the antibodies and the toxicity determinants destroyed by the modification are not necessarily identical [38,41].

As published by Bertiller et al. [36], the category of masked mycotoxins includes both extractable conjugated and non-extractable (bound) varieties. So-called “bound mycotoxins” are covalently or non-covalently connected to polymeric carbohydrates or proteins. Extractable conjugated mycotoxins can be detected by suitable testing procedures if analytical standards are available and their structure is known. On the other hand, bound mycotoxins are not directly accessible and must be released from the matrix by chemical or enzymatic treatment prior to chemical analysis. Tests for modified mycotoxin detection are generally based on water or acetonitrile extraction followed by liquid chromatography-tandem mass spectrometry (LC-MS/MS) analysis. Both targeted and non-targeted methods are utilized. These authors also confirm that immunochemical methods are not selective enough for modified mycotoxins. There is great variability in fumonisins and their entrapped forms due to different extraction strategies. Based on the lack of commercially available calibrants and reference materials, properly validated routine methods are missing [36]. Only standard solution of deoxynivalenol-3-glucoside (D3G), a derivative of DON, is commercially available [37].

### 4.2. Multitoxin Detection

A fundamental problem in detecting multitoxins is the question of which toxins should be the focal point of these tests. The prime movers for routine mycotoxin testing are applicable regulatory obligations or trade specifications. The unfavorable circumstance is the lack of globally harmonized regulations. On the other hand, variable food consumption patterns in different regions of the world should also be taken into account, so that a uniform approach may not be appropriate everywhere. The food supply chain is highly dynamic and has a large number of variables. Apparent climate change is also contributing by causing variations in fungal populations and, thus, in mycotoxin patterns. In addition, new toxins are emerging, and forerunners and metabolites of familiar toxins (masked or modified forms) are increasingly being found. The outcome is that the number of mycotoxins considered relevant increases over time [60].

Greater efficiency in hazard detection and lower monitoring expenses have the potential to enable more comprehensive surveillance, further lowering the potential for exposure—a so-called positive feedback loop. Another advantage of assays for many compounds is that they provide prevalence data that can be used as a criterion for adding a new substance to the list of those for which targeted analysis is conducive. Some of the challenges of multiple mycotoxin detection, independent of the analytical technique, are the need for co-extraction of multiple analytes with very different polarities and the potential for carryover of matrix components that can affect the results. For chromatographic tests, the challenge is to select satisfactory calibration systems. Assays based on molecular recognition are, by their very nature, subject to conditions that alter the recognition event. Moreover, while many technologies have been developed to detect molecular interactions, each has an intrinsic technological weakness that makes it challenging to use. So, there is also no ‘perfect’ immunoassay solution for multiplex detection of mycotoxins, but methods are improving, and there is good reason to believe that these challenges can be overcome with the development of convenient methods [60].

### 4.3. Future Analytical Prospects

New methods and protocols for mycotoxin detection are permanently being developed [32,61,66], and certain imminent technologies are often discussed, such as proteomics and genomic methods, molecular techniques, electronic noses, aggregation-induced emission dyes, quantitative NMR, and hyperspectral imaging [67]. Despite the fact that such a large number of analytical techniques are permanently validated and optimized and that many new methods are emerging, the LC/MS-MS technique is the principal tool for multiple mycotoxin testing. However, there are factors that limit the widespread use of chromatography, such as expensive equipment, necessary highly trained personnel, and complicated protocols for sample preparation. Therefore, the use of these methods is limited in those situations where rapid on-the-spot analyses are required, i.e., in the field, which are cases in the facilities of importers, traders, and food and feed companies. Accordingly, if there is a need for rapid determination of mycotoxins, in terms of screening, methods based on immunoassays are used [67].

The future development of analytical methods is based on effectively overcoming critical points and continuously improving the performance of the methods and techniques to obtain high accuracy and sensitivity. Crucial steps are the extraction procedure, the purification process, and the chromatographic separation, which should be highly adjusted in order to significantly reduce matrix effects. The structural modification of mycotoxins involves changing the chromatographic parameters and even the extraction efficiency, so special attention is needed in the steps of extraction and cleanup. In addition, insoluble structures cannot be detected without a treatment that makes them soluble [37]. Besides underestimation, inadequately pretreated samples can affect the sensitivity of mycotoxin detection and contribute to an overestimation of the results, as matrix effects can interfere with the interpretation of the results, especially in immunoassay-based methods with color compounds. Both the matrix and the analyte determine the outcome, so the use of HPLC after immunoaffinity purification should be validated for each mycotoxin/matrix conjunction.

High-performance liquid chromatography coupled with mass spectrometry is an essential technique to identify new metabolites and quantify the total mycotoxins in food and feed. Further improvement goes in the direction of developing extraction and cleanup strategies in order to detect and identify as many derivatives as possible for a more precise determination of the risk that food and feed can represent. As emphasized by Freire and Sant’Ana [37], direct injection techniques and the use of enzymes in more complex matrices seem to be the most promising options. Additionally, it is necessary to produce standards for these newly discovered metabolites, which, besides pure identification, will also provide precise quantification in order to enable adequate risk analysis. The lack of decisive toxicodynamic and toxicokinetic studies, along with the scarce understanding of the modification mechanisms of mycotoxins, burdens the determination of the maximum tolerable levels of these metabolites in food, which is an ultimate demand.

In connection with mycotoxicology food and feed monitoring, increased financial hindrances arise. This includes not only the direct testing costs but also the expenses incurred by manufacturers of goods whose products lose value when toxins are detected. Therefore, business decisions should be based on the content of those compounds for which consensus on the potential hazard is present and for which regulations have been issued [60]. Therefore, an appropriate and complete toxicity assessment for all mycotoxins and mycotoxin forms present in food and feed is important for the correct determination of the realistic health risk possibly triggered by the potential interaction of different conformations or groups of mycotoxins. For that reason, the extension of current multitoxin tests should be highly prioritized, as should the inclusion of newly discovered transformation products or types of mycotoxins, bearing in mind relevant needs and risks.

## 5. Conclusions

Even though the existing mycotoxin management strategies are effective, there is significant potential for their improvement, especially considering the challenges we face in practice. Additional effort should be implemented to assess the occurrence of modified mycotoxins and their toxicity, as such data are still limited. Furthermore, general mycotoxin regulations focusing on major toxins are currently based on the toxicity of individual mycotoxins, meaning that they do not consider the potential additive or synergistic toxicity of combined mycotoxins. Also, stronger efforts should be implemented to develop predictive models that encompass contamination from a wider spectrum of mycotoxins, especially taking climate change into account. Also, there is a constant need to develop new detection and decontamination strategies in accordance with effective mycotoxin risk management. Overall, mycotoxin risk management should represent the incorporation of approaches that involve risk assessment taking into account the different forms and combinations of these substances, the regular and extensive monitoring of food and feed, the periodic revision of regulatory limits in accordance with new knowledge and practical needs, as well as continuous work on developing methods to mitigate contamination.

## Figures and Tables

**Table 1:** Presence of multiple mycotoxins—literature summary.

Co-Occurring Toxins	Matrix	Incidence of Multiple Contamination [%]	Source
FUMs, DON, ZEA, NIV, T-2/HT-2	maize, wheat, barley, oats, etc.	54.9	[19]
OTA, ZEA, DON	cereals	75	[20]
ZEA, DON, NIV	wheat	74 and 12	[21]
DON, DON3G NIV, NIV3G	market foods	49	[22]
FUMs, DON, NIV, ZEA	commercial cereals	3 for quadruple 22 for triple 30 for double	[23]
DON + AFs AFs + FUMs + DON AFs + FUMs + ZEA + DON	maize	100 for double 87 for triple and more	[24]
T-2, citrinin, BEA, DON	market cereals	50 for triple and more	[25]
DON, ZEA, FUMs o rFUMs + AFB1	feed and feed materials	64	[27]
FUMs with type B trichothecenes	feed and feed materials	75	[28]
DON, ZEA, T-2/HT-2, FUMs	feed	up to 90	[29]
ENN B, DON, ZEA	cereals	42	[30]
Up to 16 analytes	pet food	99	[31]
BEA + ENNs	feed and feed materials	47	[33]
More than 5 *Fusarium* mycotoxins	maize silage	87	[34]

**Table 2:** Modified mycotoxins—most often identified according to Angioni et al. [39].

Parent Mycotoxin	Modified Mycotoxin	Common Matrix
Deoxynivalenol (DON)	DON3G; DON, 3—β-D-glucopyranoside; DON 3-acetyl; DON 15-acetyl; DON glutathione (DON-GSH); sulfo-conjugates	corn, wheat, oats, rye, barley, beer, breakfast cereals and snacks
Zearalenone (ZEA)	ZEA14G; ZEA 4-β-D-glucopyranoside; ZEA14ßDG; ZEA14ßDGp; ZEA 2,4-O-β-diglucoside; ZEA14S; palmitoyl ZEA	Wheat bran, corn-based food (grain bread, bakery products, breakfast cereals); vegetable oils
Nivalenol (NIV)	NIV3G; NIV 15-acetyldeoxy; NIV 3-acetyldeoxy; NIV 4-acetyl; NIV 4,7-dideoxy	wheat and corn
Fumonisin (FUM)	N-acyl-HFB1, HFB1	corn
T-2/HT-2	T-2-3-glucoside; HT-2-3-glucoside	oats and wheat
Neosolaniol (NEO)	neosolaniol-glucoside (NEO-G)	corn
Diacetoxyscirpenol (DAS)	diacetoxyscirpenol-glucoside (DAS-G)	corn
Fusarenon-X (FUSX)	fusarenon-X–glucoside (FUSX-G)	wheat
Ochratoxin A (OTA)	(4R)- and (4S)-4-hydroxy-OTA (4R-OH-OTA, 4S-OH-OTA); and β-glucosides (OH-OTA-β-Glc)	corn, wheat, soybean, tomato, potato, carrots, and paprika

**Table 3:** Extraction of mycotoxins (Adapted from Agriopoulou et al. [67]).

Methods	Solvents	Benefits	Limitations	Source
Quick Easy Cheap Rough and Safe (QuEChERS)	Organic solvents or mixtures (acetonitrile, methanol, methanol/acetonitrile)	Fast, simple, economical, sensitive, better reproducibility and accuracy	Need for an additional enrichment step	[72,73]
Accelerated Solvent Extraction (ASE) ꞊ Pressurized Liquid Extraction (PLE)	Organic solvent mixture (methanol/acetonitrile, acetonitrile/water)	Faster extraction, fully automated, minimal solvents, higher extraction efficiency	Costly instruments, matrix components excessively coextracted	[58,74]
Solid–Liquid Extraction (SLE)	Organic solvent mixture with water or diluted acids	Less solvents	Matrix effects	[68,75]
Liquid–Liquid Extraction (LLE)	Organic solvent mixture (hexane, cyclohexane) with water or diluted acids	Fit for small-scale preparations	Time-consuming, glassware may absorb the sample	[76]
Supercritical Fluid Extraction (SFE)	Use of CO_2_ under critical conditions, methanol, ethanol, acetone	Fast, uses smaller solvent volumes, preconcentration effect, suitable for extraction of temperature-sensitive analytes	Specific and very costly equipment, not suitable for routine analysis	[58,75]
Microwave-Assisted Extraction (MAE)	Aqueous solution (organic solvents)	Less time and less solvent usage	Expensive instruments, applicable only for thermally stable compounds	[77]
Vortex-Assisted Low-Density Solvent-Microextraction (VALDS–ME)	Mixture of organic solvents, dispersive solvents, and water	Fast, simple, effective, low-density solvent usage	Optimization after controlling a lot of parameters	[78]

## Data Availability

Not applicable.
